# Discovery of the Cryptic Sites of SARS-CoV-2 Papain-like Protease and Analysis of Its Druggability

**DOI:** 10.3390/ijms231911265

**Published:** 2022-09-24

**Authors:** Yue Qiu, Qing Liu, Gao Tu, Xiao-Jun Yao

**Affiliations:** Dr. Neher’s Biophysics Laboratory for Innovative Drug Discovery, State Key Laboratory of Quality Research in Chinese Medicine, Macau Institute for Applied Research in Medicine and Health, Macau University of Science and Technology, Taipa, Macau, China

**Keywords:** COVID-19, SARS-CoV-2 PLpro, MixMD, Probeview, TRAPP, druggability, cryptic pocket

## Abstract

In late 2019, a new coronavirus (CoV) caused the outbreak of a deadly respiratory disease, resulting in the COVID-19 pandemic. In view of the ongoing pandemic, there is an immediate need to find drugs to treat patients. SARS-CoV-2 papain-like cysteine protease (PLpro) not only plays an important role in the pathogenesis of the virus but is also a target protein for the development of inhibitor drugs. Therefore, to develop targeted inhibitors, it is necessary to analyse and verify PLpro sites and explore whether there are other cryptic binding pockets with better activity. In this study, first, we detected the site of the whole PLpro protein by sitemap of Schrödinger (version 2018), the cavity of LigBuilder V3, and DeepSite, and roughly judged the possible activated binding site area. Then, we used the mixed solvent dynamics simulation (MixMD) of probe molecules to induce conformational changes in the protein to find the possible cryptic active sites. Finally, the TRAPP method was used to predict the druggability of cryptic pockets and analyse the changes in the physicochemical properties of residues around these sites. This work will help promote the research of SARS-CoV-2 PLpro inhibitors.

## 1. Introduction

In late 2019, a new coronavirus (CoV) caused the outbreak of deadly respiratory disease, and the COVID-19 pandemic brought major harm and challenges to more than 200 countries and regions around the world [1,2,3]. Due to the profound impact of these viral outbreaks on public health and the economy, there is an immediate need to find drugs to treat patients. SARS-CoV-2 critically relies on the activity of viral proteases [4,5,6]. It has a spike protein responsible for binding to its host cell-surface receptor, angiotensin-converting enzyme 2 (ACE2) [7,8]. After entering the cell, the viral RNA attaches to the host ribosome to produce two kinds of multiple proteins, the main protease (Mpro, also known as 3CLpro, the protease domain of nsp5) and the papain-like protease (PLpro, the protease domain of nsp3), which are necessary for the production of new mature virions [9,10,11]. These proteases generate a functional replicase complex and enable viral spread, therefore they are attractive targets for antiviral therapies such as small-molecule inhibitors [12,13,14]. Viral proteases have been identified as promising targets for inhibiting the replication of viruses of diverse families, such as Coronaviridae, Flaviviridae, Retroviridae, and Picornaviridae [15]. PLpro is a relatively unusual cysteine protease that is resistant to blockade by such inhibitors [16].

**Figure 1:**
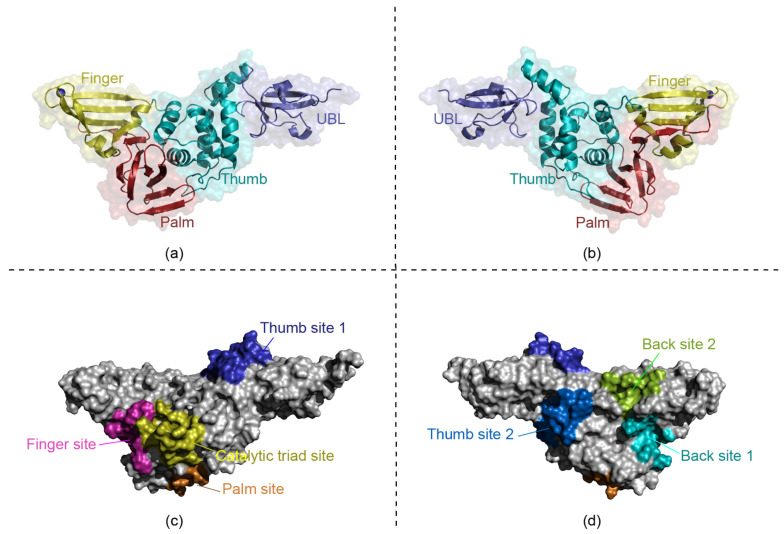
Domain division of the SARS-CoV-2 PLpro structure: (**a**,**b**) show the division of domains of SARS-CoV-2 PLpro. The blue is the UBL domain (residues 1–60), the cyan is the thumb domain (residues 61–180), the yellow is the finger domain (residues 181–238), and the red is the palm domain (residues 239–315). (**c**,**d**) show the locations of the main sites mentioned in the article, including the catalytic triad site, thumb site 1, thumb site 2, palm site, back site 1, back site 2 and finger site.

Inhibitors targeting SARS-CoV-2 PLpro are excellent candidates for antiviral drug development, as they not only block virus replication but also inhibit the dysregulation of signaling cascades in infected cells [17]. Understanding SARS-CoV-2 PLpro substrate specificity, structure, and mechanism would greatly facilitate the development of effective PLpro inhibitors by enabling rational design and research on drug retargeting. Because it is a beta coronavirus, the function and structure of SARS-CoV-2 papain-like protease (PLpro) are similar to those of SARS-CoV and MERS-CoV. The structure of the PLpro monomer comprises four distinct domains, including an N-terminal ubiquitin-like (UBL) domain and an extended right-hand architecture with “thumb–palm–fingers” [16,18]. This arrangement is similar to that of ubiquitin-specific proteases (Figure 1).

According to previous literature reports and information from the Protein Databank (PDB), there are several known SARS-CoV-2 PLpro targets, a canonical cysteine protease catalytic triad site (PDB ID: 6WUU, 7CJM) [19,20,21], a thumb region binding site (PDB ID: 7OFS), and a lower palm region binding site (PDB ID: 7M1Y). We named them the catalytic triad site, thumb site 1, and palm site (Figure 1c). However, at present, only naphthalene molecules and GRL0617 show high inhibitory activity; however, it still needs to be further optimized [19,22]. There are also no SARS-CoV-2 PLpro inhibitor drugs with good activity in clinical trials. An important reason is that the biological activity and targeting selectivity of existing inhibitors are not strong enough. The crystal structure and molecular docking results of SARS-CoV-2 PLpro showed that the shear active pocket is in the palm domain, and the pocket is shallow, which leads to poor druggability [4,23]. However, the protein has multiple zinc ions with unknown functions, which are stably bound to the region outside of the known pocket [4]. Moreover, the cryptic pocket in PLpro was also mentioned in Balkrishna A’s article [24]. These indications suggest that there may be unknown cryptic activating binding sites on the surface of PLpro. Cryptic sites are defined as sites that form pockets in ligand-binding structures but not in unbound protein structures [25]. In addition, because PLpro is only part of the shear activity of NSP3 encoding nonstructural protein NSP3, PLpro may play a role more in protein interaction with other parts of NSP3 [26,27]. Cryptic pockets often appear in the dynamic process of these interactions [28]. The cryptic pockets that have no direct relationship may also have an allosteric relationship with the active pocket, which can be used as the target of allosteric inhibitors [29]. The molecular binding mechanism of SARS-CoV-2 PLpro activation sites is unclear, which delays the development of related drug molecules. More detailed molecular simulation data will be helpful for drug design. Therefore, it is necessary to carry out molecular simulation research on PLpro and analyse its active sites to determine whether there is the possibility of other cryptic activating binding pockets.

The set of amino acid residues around a binding pocket determines its physicochemical characteristics and, together with its shape and location in a protein, defines its functionality. The mobility of proteins allows the opening, closing, and adaptation of binding pockets to regulate binding processes and specific protein functionalities [30]. Many proteins have small-molecule binding pockets that are not easily detectable in the ligand-free structures. These cryptic sites require a conformational change to become apparent [25]. Cryptic pockets appear with the appearance of protein conformation and specific dynamic changes. These changes include the rearrangement of residue side chains, the movement of the loop, the relative displacement of the domain, and so on [28]. The cryptic pocket is difficult to capture by experimental means such as X-ray crystal diffraction. However, the computational biology method can simulate the conformational changes of proteins on different time scales at the atomic level to identify and characterize cryptic binding sites [28]. Therefore, this study uses computational biology to find the cryptic pockets on the surface of SARS-CoV-2 PLpro, analyse the properties of the changes in residues around these active sites, and provide important target information for the design of subsequent small molecule inhibitor drugs.

**Figure 2:**
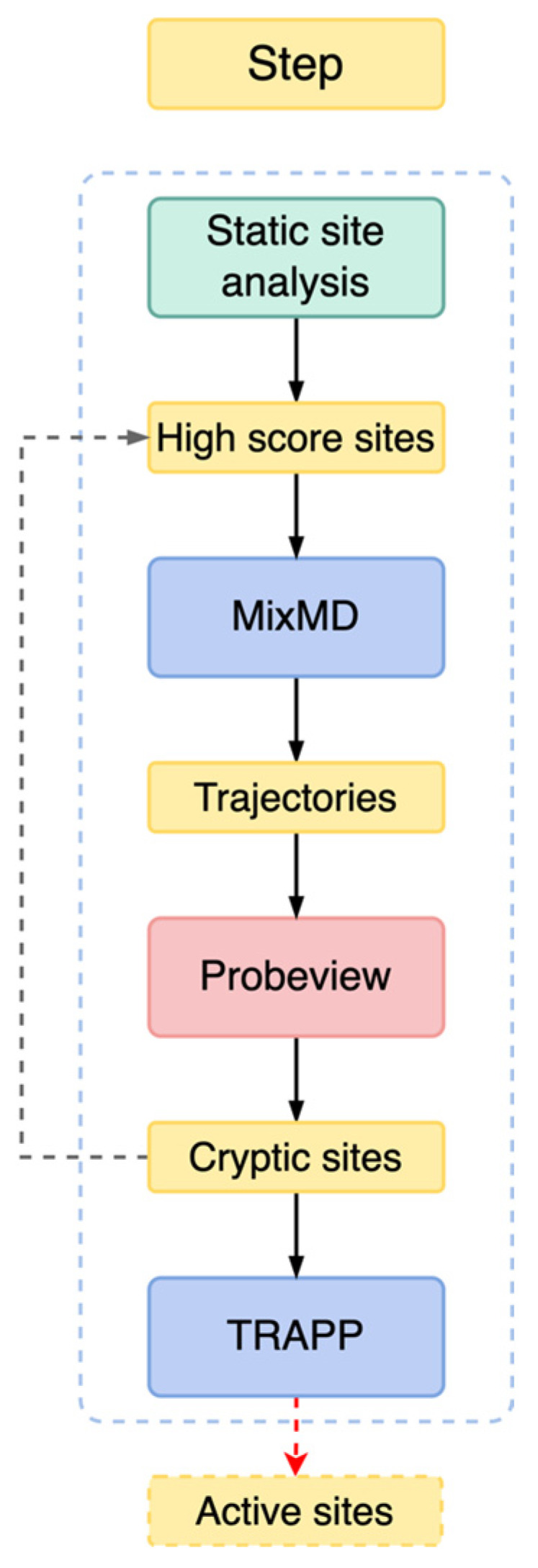
The flow of this research work: (1) Static site detection. (2) Dynamic simulation of mixed solvents. (3) Density sequencing and statistics of probe molecules. (4) TRAPP analysis.

The research workflow is shown in Figure 2. First, we detected the possible active sites of PLpro series proteins (Table 1) by three static site analysis methods: the sitemap of Schrödinger (version 2018, LLC, New York, NY, USA), the cavity of LigBuilderV3 module, and the online tool DeepSite. In Schrödinger, the sitemap module can treat entire proteins to locate binding sites whose size, functionality, and extent of solvent exposure meet user specifications [31,32]. In LigBuilder V3, the cavity module can detect and analyse the ligand-binding site of the target protein and estimate the drugabbility of the binding site [33]. In addition, we also used an online web tool, DeepSite, which is a protein binding pocket predictor based on deep neural networks [34]. One reason for choosing these conformations is that they are bound conformations. These conformations have ligands at known sites, such as 6WUU with the VIR250 in the catalytic triad site [6], 7CJM with ligands at catalytic triad sites, 7M1Y with ligands in the palm region, and 7OFS with ligands in the thumb region. The other reason was to compare whether there were some differences by detecting mutant PLpro, C111S mutant 7D47, and C112S mutant 7D6H. Through these three static site analysis methods, we verified the known binding sites and detected the possible cryptic sites to provide a reference for subsequent experiments. Second, we established the PLpro pure water system using normal molecular dynamics (MD) and five kinds of mixed solvents systems using mixed solvents molecular simulation (MixMD). In order to fully research the induction of small molecule probes and detect all cryptic pockets as much as possible. In MixMD, 5% aqueous solutions of five hydrophobic molecular probes, namely, isopropanol (IPA), acetonitrile (ACN), pyrimidine (PYR), phenol (PHN), and N-methylacetamide (NMA), were used to detect the cryptic pockets on the surface of PLpro [35,36]. We selected these probe solvents because they allowed us to capture hydrophilic, hydrophobic, hydrogen-bonding, and aromatic interactions [29,37]. In addition, NMA is the smallest molecule with peptide bonds, which can simulate the role of peptide substrates in cryptic pockets [36]. The molecular weight and volume of these probe molecules are small enough to be easily placed into the pocket as a whole, therefore they have high single atom interaction efficiency. Following simulation, for the mixed solvent system trajectory given by MD simulations, the CPPTRAJ grid command from Ambertool 21 [38] and Pymol-MixMD Probeview [39] were used to calculate and rank the occupation density of probe molecules on the surface of SARS-CoV-2 PLpro. MixMD Probeview incorporates two analysis procedures: (1) identifying and ranking whole binding sites and (2) identifying and ranking local maxima for each probe type. According to the ranking information and comparing the results of previous site detection, we can try to determine the location of possible cryptic binding sites. Reliable cryptic site prediction requires not only molecular dynamics and the pocket shape, but also the combined use of residue physicochemical properties as features of machine learning algorithms. TRAnsient Pockets in Proteins (TRAPP) is a tool that allows for the exploration of different protein conformations, the analysis of binding pocket flexibility and dynamics, and the extraction of spatial and physicochemical information on the binding pocket conformations [40]. TRAPP provides two machine learning models, a logistic regression model (TRAPP-LR), and a convolutional neural network model (TRAPP-CNN). TRAPP-LR provides a linear model for pocket druggability trained with logistic regression using global descriptors of the binding pockets (such as the pocket volume and pocket hydrophobicity). TRAPP-CNN uses a convolutional neural network to process a spatial grid representation of the properties of the binding pockets [40]. Finally, we used the TRAPP method to analyse the known sites and further judge the possible cryptic binding sites, as well as the various druggability-related attributes of the residues around the site.

## 2. Results and Discussion

### 2.1. Target Analysis Based on the Original SARS-CoV-2 PLpro Conformation

**Figure 3:**
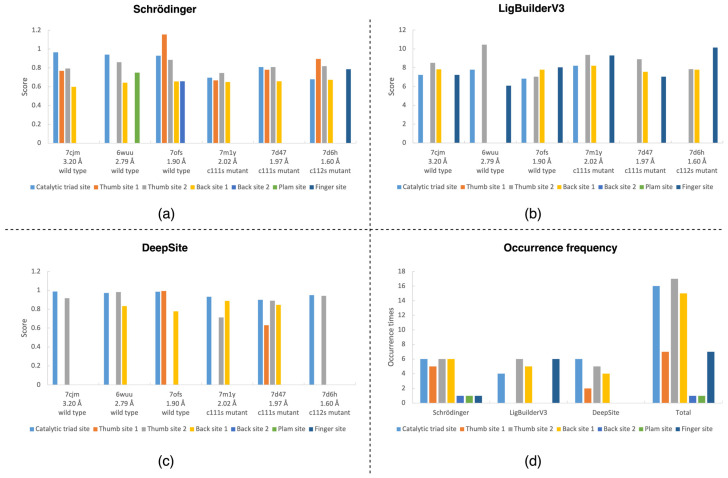
Site detection location map and main sites score statistics: (**a**) Schrödinger’s sitemap results. (**b**) Cavity of LigBuilderV3 test results. (**c**) Binding site detection results of DeepSite. (**d**) Statistics of the detected number of main sites.

Through the three static site detection methods, namely, Schrödinger, LigBuilderV3, and DeepSite, we obtained the following results. The site detection and scoring of different conformations by the three methods are shown in Figure 3a, Figure 3b, and Figure 3c, respectively. The specific scores, and other physical and chemical properties, are shown in Table S1, Table S2, and Table S3, respectively. In addition, for the convenience of observation, we present statistics on the frequency of the detected occurrence of these major sites (catalytic triad site, thumb site 1, thumb site 2, palm site, back site 1, back site 2, and finger site), as shown in the bar chart of Figure 3d. Through these results, firstly, we verified the known SARS-CoV-2 PLpro targets, catalytic triad site, thumb site 1, and palm site, as shown in Figure 1c. With these three detection methods, the known catalytic triad sites can be detected in most conformations. The known thumb site 1 was mainly detected in all conformations except 6WUU by the Schrödinger method and in the 7ofs conformation by the DeepSite method. In other cases, the thumb site 2 did not appear in the top ranking. According to the mechanism of cryptic pocket [25], we speculate that the druggable cavities of this position may be easily detected only under the induction of small drug molecules. However, the another known palm site locus appeared less frequently and scored lower. Secondly, we also found several areas where cryptic sites may exist, such as the thumb, finger, and back of the PLpro region. We named two of them, thumb site 2 and back site 1, as shown in Figure 1d. Notably, the scores of these cryptic sites are not low, and the ranking of these known binding sites is not the highest. The back site 1 and thumb site 2 were detected in many cases, and the scores ranked high, as shown in Figure 3, indicating that these two positions are likely to have cryptic active sites. Then, the detection results of different conformations are also different, and some conformations have sites that other conformations do not have. The conformational changes will cause site changes, even subtle changes. To summarise, all these methods have found the known sites, indicating that the software is reliable. In addition, some potential sites were also found. The early stage analysis of PLpro sites provides an important reference for subsequent research.

### 2.2. Search for Cryptic Sites of SARS-CoV-2 PLpro Based on Probe Molecular Density Sequencing

**Figure 4:**
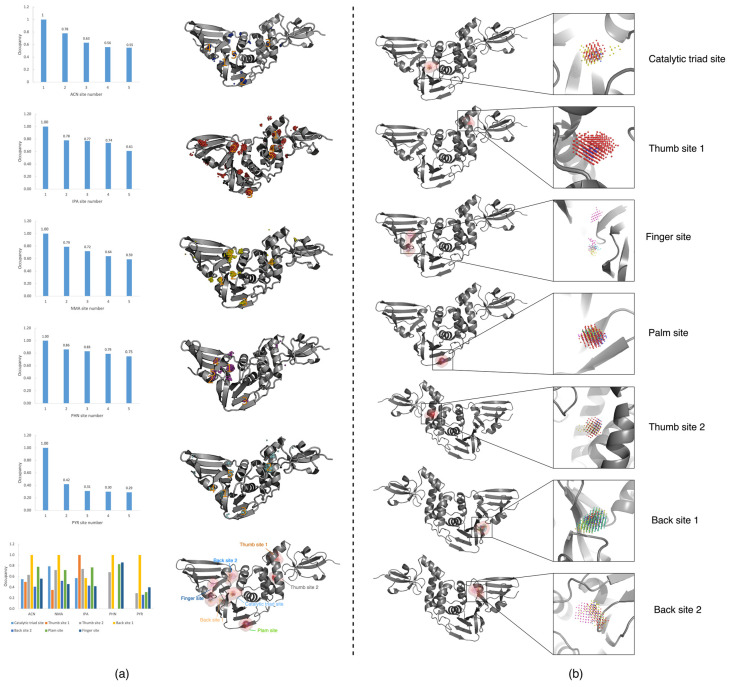
Probeview results: (**a**) Density ranking and location of sites in the PLpro MixMD maps. From top to bottom are ACN, NMA, IPA, PHN, PYR MixMD system density ranking and statistics of the scores of the main sites. (**b**) Distribution of probe molecules at the main sites. The occupancy points are coloured to different probes: ACN (blue), IPA (red), NMA (yellow), PHN (purple), and PYR (cyan).

We used MixMD Probeview to analyse the occupancy grids and obtained the hotspot ranking (Figure 4). The first five positions were selected as candidate sites. According to previous studies, for each system, the top-ranked site was either the active or allosteric site [29,39]. In the ACN MixMD system, ACN probe molecules can effectively capture hydrophilic interactions, in which the nitrogen atom is the main hydrogen bond donor. In the probe arrangement (Figure 4a), back site 1 has the most ACN distribution, followed by the known palm site. The third place is known thumb site 2. This site has appeared many times in the previous site detection and has a high score, which is likely to be a cryptic binding site. Then, the fourth and fifth positions are in the finger region and the known catalytic triad site region, respectively. Through the arrangement of probe molecules, we know that the pockets of these sites have a certain hydrophilic interaction.

In the NMA MixMD system, NMA probe molecules have the smallest peptide bond and can simulate the role of peptide substrates in cryptic pockets. In the probe distribution density ranking (Figure 4a), the first point is also distributed in back site 1. This position is consistent with the position with the highest molecular density of the ACN probe. The second point is the known catalytic triad site region, and NMA also ranks the highest in the density of the catalytic triad site among all probe molecules. The third point is another known palm site. The fourth point is thumb site 2, which has a high distribution similar to ACN probe molecules. Finally, the fifth highest density position is located above the finger of PLpro. In the previous site detection, the score of this position is not high, and there is not much distribution of probe molecules except NMA.

In the IPA MixMD system, IPA probe molecules can easily capture small hydrophobic interactions and provide hydrogen bond receptors and donors. In the molecular density ranking of the probe (Figure 4a), the first point is in the region of known thumb site 1. According to this result, we speculate that thumb site 1 has a role in the sensitive hydrophobic interaction, which is an important factor affecting the druggability of the active pocket. The second point is in the finger region; however, this region was not scored prominently in the previous site detection. Then, there is a third point, located in the region of the known palm site, followed by the fourth point, located in the thumb site 2 region mentioned above. The last point is located outside the thumb region.

For the PHN MixMD system (Figure 4a), PHN probe molecules are six-membered aromatic (less soluble) and can capture aromatic interactions very well. In this system, the first point of the PHN probe molecular arrangement is located at the back of the finger region of PLpro. This region also has the distribution of NMA probes, which is named back site 2 for the convenience of subsequent naming. However, it should be noted that numbers two, four, and five in the density ranking are in the finger region (Figure 4a), and there are a large number of PHN probe molecules in this region. Due to the problem of clustering, the ranking is not the highest. The finger region strongly attracts PHN probe molecules, indicating that this region is likely to have cryptic active sites with a strong aromatic effect, which needs further investigation. In the follow-up, we conducted TRAPP analysis on this region to verify whether this region has good druggability. The third position is in the palm area, which is a hot spot area, and the other four probes are also distributed.

For the PYR MixMD system (Figure 4a), PYR probe molecules are six-membered aromatic (more soluble) and can also capture aromatic interactions. In this system, the site with the highest molecular density of the probe is located at the back of PLpro, which is back site 1, as mentioned above. This position is also the site with the highest molecular density of NMA and ACN probes. In view of the high attractiveness of this region to all three probe molecules, it is plausible that this region has cryptic sites. Subsequently, we also analysed the back site 1 region using the TRAPP method. The second place was located in the lateral region of the thumb of PLpro, and the third place was the region of the known palm site. The fourth position is located in the finger region, which is speculated to be due to the aromatic interaction in this region, therefore it also attracts PHN probe molecules. The last site is located in the back site 2 region mentioned above, which is also the region with a high distribution of PHN probe molecules. It can be concluded that the back site 2 region also has a certain aromatic interaction, thus it can attract PHN and PYR probe molecules here. However, in the previous site detection, the score of back site 2 was not high, and the druggability was poor.

We found that there were many overlapping points in the simulated trajectories of different mixed probe molecules. We counted and ranked these highly overlapping points (Figure 4), include the known sites, namely, the catalytic triad site, thumb site 1 and the palm site, as shown in Figure 4b. In the region of the catalytic triad site, there are three types of probe molecular distributions, ACN, IPA, and NMA, of which NMA has the highest molecular density. In the region of thumb site 1, there are also three kinds of probe molecules; however, they are mainly IPA probes, followed by some ACN and very little NMA. In the region of the known palm site, there are five probe molecules distributed in this site region, but they are not the highest in their respective rankings. However, due to its shallow pocket, the score in the previous site detection is not high, and, through molecular docking, the score of docking in this region is low (it was scored by Schrödinger SP docking). Although it is a known site with known complex crystals, 7M1Y, due to the poor analysis and scoring results, it is speculated that the binding activity of this site is poor, and we have excluded the follow-up study of this site. In addition, there are some possible unknown binding sites, such as thumb site 2, finger site, back site 1, and back site 2, as shown in Figure 4b. In the region of thumb site 2, there are four kinds of probe molecular distributions: ACN, IPA, NMA, and PYR. Except for a lower PYR distribution, the other three have a high-density arrangement. As mentioned earlier, this position is likely to be a cryptic site with high activity. For the finger site, five probe molecules are distributed here, mainly PHN and PYR aromatic probe molecules, followed by some NMA molecules and a small amount of IPA and ACN. According to the previous results, this site is likely to be an aromatic cryptic binding site. At back site 1, the probe molecules of the three systems ranked first in their density, ACN, NMA, and PYR, followed by some IPA distribution. This region site is also a possible cryptic site. In the region of back site 2, there are also five kinds of probe molecular distributions, mainly PHN and PYR, as well as a certain number of NMA, a small number of IPA, and very little ACN. Although this site can attract aromatic probe molecules PHN and PYR, and it is among the top five in the probe molecular density ranking of both systems, it is likely to be a binding site with high activity according to the above site analysis with three software programs. In summary, in these high overlapping point sites, for palm and back site 2, due to the low scores of the sites in the corresponding region in various site detection methods and the poor results of molecular docking found in previous studies, we speculate that it is due to the shallow pocket in the corresponding region, which leads to poor druggability. Therefore, we excluded the possibility of cryptic sites with high binding activity in the palm and back site 2 regions and did not conduct subsequent residue analysis. Compared with the previous site analysis of the original structure, we finally identified the three most likely cryptic sites: back site 1 and finger site and thumb site 2. Then, we used the TRAPP method to analyse the residues around the three cryptic sites and the previous two known sites, catalytic triad site and thumb site 1.

### 2.3. Analysis of Six Model Systems MD Results

**Figure 5:**
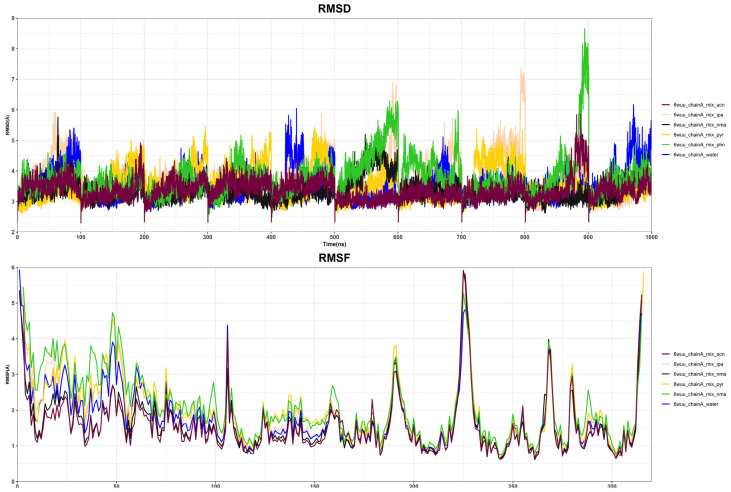
Changes in RMSD and RMSF values in 6 simulation systems.

We simulated the mixed solvent molecular dynamics, established the solvent system of five mixed probe molecules, attempted to use five probe molecules to induce the conformational change of PLpro, and then attempted to locate the cryptic active pocket. In this study, the stability of six model systems was verified according to the molecular dynamics trajectory of the simulated system. The root-mean-square fluctuation (RMSF) of each amino acid residue and the root-mean-square deviation (RMSD) between the amino acid heavy atom near the protein main chain and the initial conformation were calculated mainly based on the analysis program of the GROMACS 2018.7 software (Figure 5). We analysed the relationship between RMSD and the simulation time to evaluate the stability of the simulation system and compare the differences in the RMSD of multiple systems. According to the initial conformation, using unbiased molecular dynamics simulation, the protein was sampled under the explicit solvent model. The RMSD results show that the protein system approached the state of convergence after 50 ns of simulation. Compared with the initial conformation, the fluctuation range of the RMSD of the whole protein skeleton atom in the simulation process was approximately 6 Å. In all systems, there was no significant difference in RMSD when each system was compared in pairs.

The RMSF results (Figure 5) showed that the regions of residues 220–240 are the most flexible, with a deviation of up to 5 Å from the reference structure. The second is the 260–270 region, in which the PYR MixMD and ACN MixMD systems can be close to 6 Å, and the other four groups can reach approximately 4.5 Å. In every system, the RMSF values of residues in most regions of the protein do not exceed 2 Å when compared with each other, therefore the conformational change of protein residues is not evident from the result of the RMSF value alone. Proteins are dynamic and possess an inherent flexibility, which alters the shape and properties of their binding pockets. The appearance of cryptic pockets often requires corresponding conformational changes in the protein. However, if the change in conformational residues is very small, conventional methods are often not sensitive enough to detect the corresponding changes, therefore it is impossible to judge the druggability of the activated binding sites. Some methods that combine pocket druggability prediction and the molecular dynamics simulation have been developed, such as MDpocket [41] and JEDI [42]. However, these methods use only a few descriptors, such as volume and hydrophobicity, and therefore might not be sensitive enough to capture the variations in druggability due to subtle conformational changes [43]. In this case, we apply the TRAPP method. The TRAPP method is designed to trace changes in the spatial and physicochemical properties of a specified pocket in a protein that may arise due to the protein’s flexibility. Therefore, to further analyse and compare the small conformational changes of residues around the active pocket of PLpro, we used the TRAPP method to analyse the druggability and physicochemical properties of multiple sites in detail.

### 2.4. Analysis and Calculation of the Pocket Druggability of Activated Binding Sites

By using the TRAPP method, we dynamically analysed these main sites: catalytic triad site, thumb site 1, thumb site 2, finger site, and back site 1. First, we used the TRAPP-LR/CNN score curve to evaluate the druggability of each site. Second, we analysed the obvious properties changes of each site. Third, we analysed the associated residues at each site. Finally, we analysed the conformation after clustering and compared it to explore the main factors affecting the druggability of this site.

**Figure 6:**
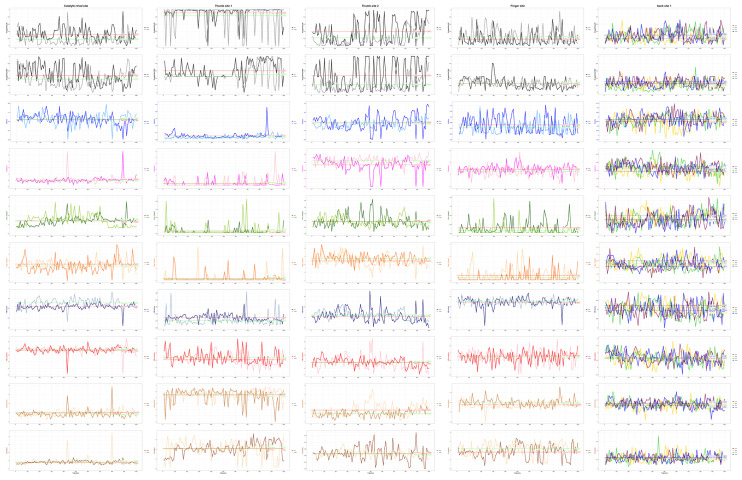
Physicochemical properties of the pocket along the MD trajectory. From left to right are the catalytic triad site, thumb site 1, thumb site 2, finger site, and back site 1.

For the catalytic triad site, according to the molecular density ranking of the probe, we selected the NMA mixed solvent system trajectory for comparison with the pure water system trajectory. It can be seen from Figure 6 that, in the scoring curves and average values of LR and CNN models, the pocket druggability of the NMA MixMD system is better than that of the pure water system. This confirmed that the induction of NMA probe molecules accelerated the druggability of catalytic triad website to a positive extent. From Figure S1A, it can been seen that the residues in Table 1 are related to the catalytic triad sites. Compared with the NMA MixMD system and pure water system, the residues associated with pockets are basically the same, and there is no obvious difference, which indicates that the insertion of the NMA probe molecules has little effect on the residues related to the catalytic triad site. In the clustering results of the NMA MixMD trajectories, we loaded cluster results in the druggability heatmap (see Figures S3A, S4A and S5A). In the LR model, the highest druggability score (0.959) occurs in Frame 9, where the hydrophobicity of the pocket (1.759), positively charged residues (−1.367), and the low number of H-bond donors (−3.516) seem to be strong contributors to the high scores. However, the CNN model score is only 0.04. The small volume of pocket (−2.527) and H-bond acceptors (1.717) were the major contributors to the low druggability score. The point with the lowest druggability score (0.018/0.057) in the NMA MixMD trajectory occurs in Frame 1, where the volume of the pocket (−2.237), high number of positively charged residues (2.002), and number of H-bond donors (2.479) seem to be a strong contributor to the lower druggability score when compared to the reference structure. In the same way, we derived the results of the pure water system for comparison (Figures S3A and S4). The highest druggability score (0.408/0.884) occurs in Frame 7, and the small number of positively charged residues (−1.238) were the major contributors. The lowest druggability score (0.038/0.051) occurs in Frame 2. The absence of H-donors (1.741) and acceptors (2.60) and the low volume of the pocket (−1.530) were the major contributors to a lower druggability score. From the above results, we know that the main factors restricting the druggability of the catalytic triad site include pocket volume, the hydrophobicity of the pocket, positively charged residues, and a number of H-bond donors. However, after the insertion of the NMA probe, the volume induction effect on the catalytic triad pocket is almost indiscernible; moreover, it only has some changes in other properties. However, from the point of view of the LR and CNN druggability curve, there is nonetheless evidence of improvement.

For thumb site 1, we selected the IPA mixed solvent system trajectory to compare with the pure water system trajectory. In Figure 6, first, the pocket LR and CNN druggability score curve of the mixed solvent system is slightly better than that of the pure water system. Second, the pocket volume score curve of the IPA MixMD system is generally higher than that of the pure water system. Third, in the hydrophobic interaction curve, after inserting IPA, it is slightly improved. This shows that IPA has some influence on thumb site 1. In the cluster results (Figure S2B), compared with the pure water system, there are more conformational types in the IPA mixed solvent system. It is also preliminarily judged that IPA has a certain impact on the residues near thumb site 1. From the results of pocket contact residues shown in Figure S1B, we found that the residues in Table 1 were mainly related to thumb site 1. Among them, the correlation of residues 56–84 is the most obvious, while the correlation of residues 127–132 and 149–156 is weak. Compared with the pure water system, the colour of residues 11, 58, 68, 69, 72, 74, 77, 79 and 80 in the IPA MixMD system is deeper, and the degree of correlation is greater, indicating that the insertion of IPA has a certain inducing effect on the formation of the thumb site 1 pocket and increases the contribution of heavy atoms in these residues. In the snapshot section (Figures S3B, S4B and S5B), Frame 4 has the highest druggability score (0.997/0.902) in the NMA MixMD trajectory of the LR and CNN models. The high hydrophobicity (4.261) was the major contributor to the high druggability. Frame 3 has the lowest druggability score (0.062/0.097), which is caused by the small volume (−2.423) of the pocket and the presence of the H-donor (3.052). In the results of the pure water system, the highest druggability score (0.994/0.470) occurs at snapshot 3. The high hydrophobicity of the pocket (4.220), the low enclosure of the pocket (−2.432), the absence of positively charged residues (−1.367), and H-bond donors (−2.265) were the major contributors. In the CNN model, the highest score is 0.902 for the IPA system, while it is only 0.470 for the pure water system. Compared with the snapshot, this result may be due to the fewer hydrogen bond receptors, the increase in hydrophobicity, and the slight increase in the pocket volume in the IPA system. These results showed that hydrophobic interactions are the main factor restricting the druggability of the thumb site 1 pocket, and the insertion of the IPA probe molecule can induce an increase in hydrophobicity and a slight increase in the volume of the thumb site 1 pocket, making the pocket druggability of the IPA system better than that of the pure water system to a certain extent.

In addition to the above two known sites, we also analysed the other three possible cryptic sites. For thumb site 2, we selected the ACN mixed solvent system trajectory for comparison with the pure water system trajectory. In both the CNN and LR model curves (Figure 6), the druggability score of thumb site 2 of the ACN MixMD system is obviously better than that of the pure water system. First, from the average value of LR and CNN, the ACN MixMD system is approximately 4, and the pure water system is only approximately 2. In addition, the trajectory score of the ACN MixMD system can reach 1.0 many times; however, in a pure water system, the maximum score can only reach approximately 0.8. In the score curve of the hydrogen bond donor (Figure 6), the influence of inserting ACN can be clearly seen. The score of hydrogen bond donors in the ACN MixMD system can reach up to 5, while the highest score of this item in the pure water system is only 2. Although the average hydrogen bond donor value is slightly lower than that of the pure water system, the appearance of high score conformations in the overall trajectory cannot be ignored. In addition, in the hydrophobic interaction and aromaticity curves, the ACN MixMD system is improved to varying degrees compared with the pure water system. Finally, the pocket volume of the ACN MixMD system also increases slightly. In the results of the pocket contact residues (Figure S1C), we found that the residues in Table 1 were mainly related to thumb site 2. Compared with the pure water system, the ACN MixMD system showed a more obvious correlation among residues in the range of 72–80, especially residues 74, 75, 76, and 77. This shows that the insertion of the ACN probe induces changes in these residues around the thumb site 2 pocket and increases the contribution of these residues to the thumb site 2 pocket to a certain extent. Then, we loaded cluster results in druggability information for the selected snapshot sections (Figures S3C, S4C and S5C). The highest druggability score (0.982/1.000) occurs in Frame 2, where the pocket volume (0.873) and hydrophobicity (0.991) of the pocket seem to be the strongest contributors to druggability. Second, the small number of positively charged residues (−0.900), H-bond acceptors (−0.991), and donors (−0.522) also has some positive influence. The lowest druggability score (0.000/0.065) occurs in Frame 7, where the low pocket volume (−2.382), more positively charged residues (1.819), H-bond acceptors (6.728), and donors (4.104) seem to be the reason for lower druggability. Compared with the water system, the reasons for the high and low scores of druggability are similar. These results indicate that the insertion of ACN probe molecules induces a change in the physical properties of the thumb site 2 pocket to a certain extent and improves the druggability of thumb site 2. Combined with the results of the previous site analysis and probe distribution, thumb site 2 has a certain development potential and needs further investigation in the future.

For the finger site, we selected the PHN mixed solvent system trajectory to compare with the pure water system trajectory. Through observing the score results curve (Figure 6), in the LR and CNN model, after inserting PHN, the druggability and aromatic curve did not improve much, or, in some instances, even decreased. Combined with the previous probe distribution, it is speculated that the finger position can adsorb a large number of PHN probe molecules, but that it has little effect on the changes in the pocket properties of the finger site. From the results of pocket contact residues shown in Figure S1D, we can see that the residues in Table 1 were related to the finger site. Compared with the pure water system, the red lines of residues 208 and 218–221 in the PHN MixMD system are more obvious, especially the colour of the residue 208, indicating that the insertion of PHN induces these residues and increases the association between these residues and finger site pockets. It is worth noting that the colour of the residues 245–250 and 298–301 is lighter than that of the water system, indicating that the contribution of these residues is even reduced due to the addition of PHN. Overall, PHN probe molecules could not obviously induce the formation of the finger pocket. In the snapshot part (Figures S3D, S4D and S5D), the highest druggability score (0.530/0.351) is at snapshot 2 in the PHN MixMD system. The high hydrophobicity (0.649) and the absence of H-acceptors (−0.885) are the major contributors to druggability. The lowest druggability score (0.027/0.071) is at snapshot 4, where the presence of H-donor (1.153), the enclosure of the pocket (2.323), and the small volume of the pocket (−1.969) seem to be the reason for lower druggability score. In the pure water system, the highest druggability score (0.455/0.212) is at snapshot 4, and the absence of positively charged residues (−1.367) seems to be the reason for the high druggability. The lowest druggability score (0.037/0.153) is snapshot 5, where a high number of H-donors (1.563), H-acceptors (2.423), and the small volume of the pocket (−1.988) seem to be strong contributors to a lower druggability score. According to the above drug availability score, even if the probe molecule is inserted, the druggability of the finger site cannot be improved, and the overall trajectory curve score of both the LR model and CNN model is low. Thus, the possibility of this site becoming a cryptic binding site is very small.

For back site 1, because the ranking of NMA, ACN, and PYR is the highest in the probe distribution, we selected these three groups of mixed solvent system trajectories to compare with the pure water system trajectory. According to the scoring of the four systems (Figure 6), from the druggability and other physicochemical properties of the pocket curves, the changes of the four systems are significantly small, and the druggability score was not improved compared with the pure water system, indicating that the insertion of probe molecules has little effect on the properties of their pockets. In the results of pocket contact residues (Figure S1E), we found that the residues in Table 1 were related to the back site 1 pocket. It is worth noting that, compared with the pure water system, residues 124, 238, 239, 279, and 280 in the other three MixMD systems showed a more obvious correlation, especially in the can MixMD system. This shows that the insertion of probe molecules does induce some residues to change; however, the change is small, and the overall impact on the back site 1 pocket is not obvious. In addition, the induction effect of the ACN probe molecules is slightly better than that of NMA and PYR probes. In the snapshot section (Figures S3E, S4E and S5E), the highest druggability score (0.678) of the LR model is Frame 1 in the NMA MixMD system, and the absence of H-bond donors (−0.678) is the major contributor to druggability. The highest druggability score (0.443) of the CNN model is at snapshot 5 in the ACN MixMD trajectory, which seems to be mainly caused by the pocket volume (0.411) and the absence of the H-donor (−0.539) inside the binding pocket. Based on all the low score snapshots, the main reasons affecting the low scoring include the enclosure of the pocket, absence of hydrophobility, and the presence of positively charged residues inside the binding pocket. The biggest impact is caused by the enclosure of the pocket, and, even if the probe molecule is inserted, this item has not been improved much. In summary, although back site 1 has strong attraction for various probe molecules, it has a low probability of being a cryptic active binding site for the above reasons.

Using the TRAPP method, we analysed the change in the physicochemical properties of the residues around each active binding site in different systems. From the overall druggability scoring curves of the TRAPP-LR/CNN models (Figure 6), the result of thumb site 1 is the best, followed by thumb site 2, the catalytic triad site, finger site, and back site 1. Among them, after inserting the probe molecule, the clearest enhancement in the druggability score was seen for thumb site 2, followed by thumb site 1, and then the catalytic triad site. Moreover, the druggability of the finger site and back site 1 is poor; therefore, we estimate that they are less likely to become cryptic sites. In terms of other physicochemical properties, for two models, the pocket volume and hydrophobicity are the main properties that are positively correlated with the druggability score, while the other global properties mostly have a negative correlation [40,43]. Through a horizontal comparison of the different sites of the scoring curves of several physicochemical properties that mainly affect the druggability of the pocket, we obtained the following analysis results. According to the average score of pocket volume and the overall volume curve, the order from high to low is thumb site2, back site 1, catalytic triad site, finger site and thumb site 1. Among them, the pocket volume score of thumb site 2 is the highest, and the peak can reach 1.5. After the induction of ACN probe molecules, there is a certain degree of improvement. This is an important reason for why we speculate that thumb site 2 may become a cryptic binding site. Although thumb site 1 is a known site, this property limits the development of its druggability, although it is slightly improved after induction by the IPA probe. According to the results of the hydrophobicity analysis, in order from high to low, is thumb site 1, finger site, thumb site 2, catalytic triad site, and back site 1. Among them, the hydrophobicity of thumb site 1 is the best, which also explains the reason why the pocket showed high hydrophobicity in the previous site detection and attracted IPA probe molecules. Moreover, after the insertion of IPA probe molecules, the hydrophobicity of thumb site 1 was slightly improved, and the average value increased from 3.646 to 3.944. According to the average score of the exposure and the overall exposure curve, the pocket enclosure score order, from high to low, is back site 1, finger site, thumb site 2, catalytic triad site, and thumb site 1. The higher pocket enclosure, the more unfavorable the druggability of the pocket. Among them, thumb site 1 is the most exposed pocket, which also explains part of the reason why it becomes a known site. In addition, it is worth noting that, after the induction of probe molecule ACN, this property of thumb site 2 has been improved to a certain extent, and the average score has decreased from 0.7019 to 0.1950. This is another important reason why we speculate that thumb site 2 may become a cryptic binding site.

In summary, for the catalytic triad site, we found a pocket volume that restricts its pocket druggability, which was not improved by the insertion of NMA probe molecules. For thumb site 1, we know that the insertion of IPA probe molecules has a positive impact on the druggability and related physical and chemical properties, and, among the five sites analysed, the result is the best from the druggability scoring curve of the overall trajectory. For the two known binding sites, through the above results, we know that the physical and chemical properties of thumb site 1 are more likely to be changed after being induced by the probe molecules to increase the druggability. However, at present, most studies mainly focus on the catalytic triad site. In the future, it may be necessary to strengthen the research on thumb site 1, hoping to find better small molecule inhibitor drugs for SARS-CoV-2 PLpro. Thumb site 2 may be the next SARS-CoV-2 PLpro potential cryptic binding site. At the same time, we also found that the insertion of the ACN probe molecules has a positive inducing effect on the improvement of the druggability of thumb site 2.

## 3. Materials and Methods

### 3.1. Static Site Analysis

The initial X-ray crystal structures of SARS-CoV-2 PLpro were downloaded from the Protein Databank (PDB), PDB ID (Table 2): 6WUU [6], 7CJM [19], 7D47 (to be published), 7D6H [44], 7M1Y (to be published), 7OFS (to be published). First, we use sitemap of Schrödinger (version 2018, LLC, New York, NY) and counted the top 5 test results. Second, we used the cavity module of LigBuilderV3 [45] to find and predict binding sites and counted the top 5 test results. Finally, we used the online tool DeepSite [34] to detect the site of SARS-CoV-2 PLpro and reserved all test results.

### 3.2. MixMD Simulations

We download the initial X-ray crystal structures of SARS-CoV-2 PLpro from the Protein Databank (PDB), PDB ID: 6WUU. The probe molecules of IPA, ACN, PYR, PHN, and PYR were obtained from Ligand Expo web (http://ligand-expo.rcsb.org/ld-search.html, accessed on 20 July 2022). First, the downloaded protein file (6WUU.pdb) was prepared by Schrödinger’s protein preparation wizard (version 2018), which removed excess water molecules and heteroatoms from the protein, built the missing side chain atoms, corrected the structure of the protein, and added hydrogen atoms. Then, we run the MD for the six systems, single water of 6WUU, and mixed solvent (5% aqueous solutions of IPA, ACN, PYR, PHN and NMA, respectively) of 6WUU with GROMACS (version 2018.7) [46]. The system molecules were described by the CHARMM27 Force Field and TIP3P water model. The temperature and pressure of the systems were maintained at 310 K and 1 atmosphere, respectively. The coordinates, energy, temperature, and other parameters of the trajectory were output once every 1 ns. The simulated step size was 2 fs. Periodic boundary conditions were used in the system. This was followed by a simulation of 100 ns. For six systems, 10 independent simulations were carried out, resulting in 1 µs of cumulative production simulation time.

### 3.3. Probeview Procedures

Following simulation, 1 µs of each MixMD trajectory was aligned and generated in a 0.5 Å × 0.5 Å × 0.5 Å grid using the CPPTRAJ module in AmberTools21. Load files to be analysed into PyMOL-MixMD Probeview. MixMD Probeview was used for the analysis of the occupancy grids and obtained the hotspot ranking and to compare the position detection results obtained in the previous target analysis and delete the pockets with low druggability.

### 3.4. TRAPP Workflow

**Figure 7:**
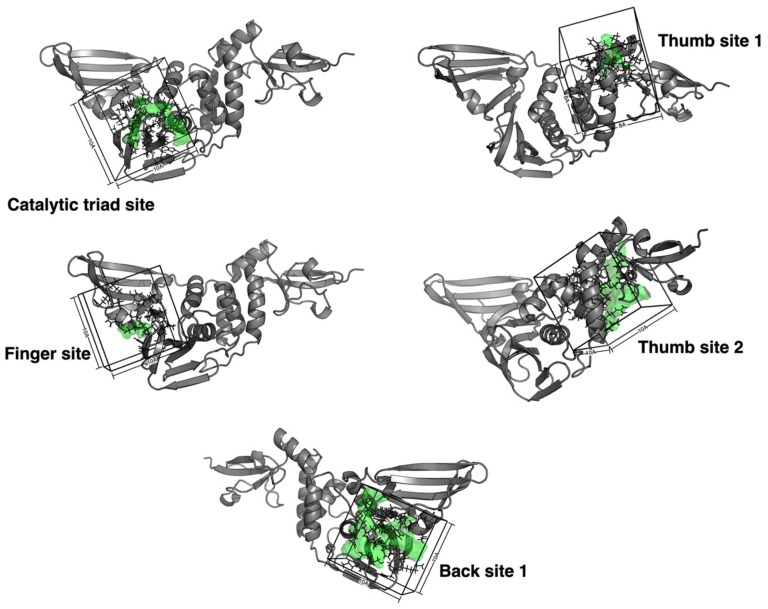
The selected residues around five sites of SARS-CoV-2 PLpro.

In the six systems, single water, and five mixed solvents, a 100 ns trajectory was taken. For different binding sites, according to the density ranking of probe molecules, we selected the probe mixed solvent system with the highest ranking at the corresponding points to compare with the pure water solvent system to observe the effect of the probe molecules on sites. According to the pocket position, the residues around the binding site are selected. We selected the residues of the catalytic triad site, thumb site 2, and finger site and back site 1 within 10 Å and the residues of thumb site 1 within 8 Å as the comparison objects (Figure 7). This provided a reference for the binding site position in a protein and is used for sequential alignment and superposition of protein structures from an ensemble or a trajectory. Then, the clustering procedure was run, backbone atoms were used for RMSD calculations, and fast hierarchical clustering of the binding site conformations was carried out with a threshold of 3 Å. Figure S2 illustrates the distribution of snapshots among clusters. Finally, the shape and physicochemical properties of the binding site region were computed for each structure. The druggability score of each frame in the MD trajectory was predicted by both the TRAPP-LR and TRAPP-CNN. Then, the pocket druggability is computed for each snapshot and plotted to show its variation. For TRAPP method, its physicochemical properties of the binding site were calculated by the following formula (Table 3). The results of the score of TRAPP-LR/CNN models and various properties changes of pocket are shown in Figure 6. The results of the pocket contact residues are shown in Figure S1 and Table 1. In Figure S1, colour defines the number of heavy atoms in each residue that contribute to the pocket boundary in a particular snapshot. The darker the colour, the closer the residue relates to the pocket. The physicochemical properties of the pocket along the MD trajectory of the clustering result are shown in Figure S3. The score heatmap of the reference conformation, the lowest score cluster, and the highest score cluster are derived, respectively, as shown in Figure S4. The pocket conformation for the reference conformation, the lowest score cluster, and the highest score cluster are derived, respectively, as shown in Figure S5.

The grid contains N grid points in one channel. A grid point is denoted as $r_{i}$, where i = 1, …, N. p represents a particular protein structure. G, $G^{ch},$ and $G^{at}$ are the distribution functions for cavity, charged atoms, and other atomic properties, respectively. The grid spacing is denoted as $l_{p}$; thus, a unit volume and a unit surface area in the grid are ${\left(l_{p}\right)}^{3}$ and ${\left(l_{p}\right)}^{2}$. $r_{i_{\left(-1\right)}}$ denotes the grid point that is examined before the current grid point $r_{i}$ in the region growing algorithm. The indicator function [·] represents a function that outputs 1 if the condition is satisfied and 0 otherwise. The set Q holds all grid points that are within the pocket.

## 4. Conclusions

Currently, the COVID-19 pandemic has become a major global public health crisis. In view of this situation, there is an urgent need to find drugs to treat patients. The papain-like protease domain of SARS-CoV-2 nsp3 has emerged as a viable drug target for the development of anti-SARS therapeutics. Primarily, through three protein site detection methods, sitemap of Schrödinger, cavity module of LigBuilderV3, and DeepSite, we verified the known SARS-CoV-2 PLpro targets, namely, catalytic triad site, thumb site 1, and palm site, and tested the reliability of these methods and found several regions that may have cryptic sites. Subsequently, through MixMD and Probeview procedures, we obtained the molecular density ranking of the probe, made site statistics for each mixed system, found some highly overlapping sites, compared the previous site analysis of the original structure, and then identified the three most likely cryptic sites: finger site, thumb site 2, and back site 1. Next, we used the TRAPP method to analyse the known sites and possible cryptic binding sites, as well as the various druggability related attributes of the residues around the site. Eventually, through a series of analyses and comparisons, we found that thumb site 2 has a certain development potential, and the in-depth study of this site will promote the development of inhibitor drugs against SARS-CoV-2 PLpro. In summary, we hope that this paper will promote drug design and discovery against COVID-19.

## Figures and Tables

**Table 1:** The related residues of the pocket boundary.

Site	Related Residues	The Most Related Residue (More than Six Heavy Atoms Associated with the Pocket Boundary)
Catalytic triad site	106–119, 162–174, 205–212, 243–251, 260–277, 297–304	110–112, 161–167, 244–248, 422 261, 263, 267–272, 301, 302
Thumb site1	10–13, 32–35, 56–84, 127–132, 149–156	56–84
Thumb site 2	10–17, 33–40, 53–59, 62–95, 125–153	12, 13, 14, 17, 56, 71, 72, 82, 83, 86, 91, 95, 130, 133, 134, 138, 142–147, 149
Finger site	206–223, 228–233, 243–253, 256–264, 297–304	208–211, 214, 218–221, 245–250
Back site 1	101, 117, 118, 121–124, 172, 177, 180, 181, 204, 209–220, 233, 236–244, 250–261, 274–283, 285, 293–296, 300–314	100, 121–124, 211–214, 217, 218, 240–242, 250–259, 277–281, 303–310

**Table 2:** SARS-CoV-2 PLpro structures.

PDB	Resol.	Released	Ligand IDs	Protein	PubMed ID	Metals
7D6H	1.60 Å	2020-11-04	PO4	NSP3: PLpro	33979649	Zn^2+^
7OFS	1.90 Å	2021-05-12	YRL	NSP3: PLpro	-	Zn^2+^
7D47	1.97 Å	2020-10-07	CA	NSP3: PLpro	-	Ca^2+^; Zn^2+^
7M1Y	2.02 Å	2021-03-24	NA, FMT, IOD, 9JT	NSP3: PLpro	-	Na^+^; Zn^2+^
6WUU	2.79 Å	2020-05-20	VIR250	NSP3: PLpro	33067239	Mg^2+^; Zn^2+^
7CJM	3.20 Å	2020-09-02	TTT	NSP3: PLpro	33473130	Zn^2+^

**Table 3:** Definitions of the global descriptors generated in the TRAPP–pocket procedure.

Pocket Property	Definition
Pocket volume	$\overset{N}{\underset{i=1}{\sum }}{\left(l_{p}\right)}^{3}\times \left[G\left(r_{i},p\right)>0\right]$
Protein-exposed surface area	$\overset{N}{\underset{i=1}{\sum }}{\left(l_{p}\right)}^{2}\times \left[G\left(r_{i},p\right)=0\wedge G\left(r_{i_{\left(-1\right)}},p\right)>0\right]$
Solvent-exposed surface area	$\overset{N}{\underset{i=1}{\sum }}{\left(l_{p}\right)}^{2}\times \left[G\left(r_{i},p\right)=-1\wedge G\left(r_{i_{\left(-1\right)}},p\right)>0\right]$
Pocket exposure	Solvent-exposed surface area/Protein-exposed surface area × 100(%)
Positively charged Negatively charged	$\underset{i\in Q}{\sum }{\left(l_{p}\right)}^{3}\times G^{ch}\left(r_{i},p\right)\times \left[G\left(r_{i},p\right)>0\right]$
Hydrogen-bond donor Hydrogen-bond acceptor Hydrophobic Aromatic Metal ion	$\overset{N}{\underset{i=1}{\sum }}{\left(l_{p}\right)}^{3}\times G^{at}\left(r_{i},p\right)\times \left[G\left(r_{i},p\right)>0\right]$

## Data Availability

The raw data supporting the conclusions of this article will be made available by the authors, without undue reservation.

## Supplementary Materials

The supporting information can be downloaded at: https://www.mdpi.com/article/10.3390/ijms231911265/s1.
