# Supplementary material for: Discovery of the Cryptic Sites of SARS-CoV-2 Papain-like Protease and Analysis of Its Druggability
Source: Int J Mol Sci. 2022 Sep 24;23(19):11265. doi: 10.3390/ijms231911265 (PMC9569941; doi:10.3390/ijms231911265)
Supplement: Supplementary file 1 [file ijms-23-11265-s001.zip › ijms-1867655-SI.pdf]

Supporting Information

Discovery of the Cryptic Sites of SARS-CoV-2 Papain-Like Protease and Analysis of Its Druggability

Yue Qiu †, Qing Liu †, Gao Tu and Xiao-Jun Yao *

Dr. Neher's Biophysics Laboratory for Innovative Drug Discovery, State Key Laboratory of Quality Research in Chinese Medicine, Macau Institute for Applied Research in Medicine and Health, Macau University of Science and Technology, Taipa, Macau, China

* Correspondence: xjyao@must.edu.mo; Tel.: +853-88972438

† These authors contributed equally to this work.

Content

Table S1. Site detection information of sitemap 2

Table S2. Site detection information of Cavity 3

Table S3. Site detection information of DeepSite 4

Figure S1. The contribution of each residue to the pocket boundary in trajectories. 5

Figure S2. The distribution of clusters. 6

Figure S3. Physicochemical properties of the pocket along the MD trajectory of the clustering result. 7

Figure S4. The heatmap for the TRAPP-LR/CNN models for selected specific cluster. 8

Figure S5. Physicochemical properties of the pocket along the MD trajectory of the clustering result. 9

Table S1. Site detection information of sitemap

Entry Name	SiteScore	size	Dscore	volume	exposure	phobic	philic
6wuu							
6wuu_sitemap_1_site_1	0.941	91	0.956	230.496	0.639	0.442	1.027
6wuu _sitemap_1_site_2	0.860	63	0.818	226.037	0.596	0.449	1.095
6wuu _sitemap_1_site_3	0.749	49	0.734	114.219	0.680	0.541	0.884
6wuu _sitemap_1_site_4	0.767	45	0.725	98.784	0.536	1.080	0.989
6wuu _sitemap_1_site_5	0.642	32	0.519	81.634	0.595	0.023	1.214
7cjm							
7cjm _sitemap_1_site_1	0.966	119	0.993	242.501	0.570	0.342	1.017
7cjm _sitemap_1_site_2	0.793	54	0.686	170.471	0.625	0.151	1.264
7cjm _sitemap_1_site_3	0.768	39	0.794	71.001	0.661	1.549	0.465
7cjm _sitemap_1_site_4	0.598	26	0.477	100.499	0.766	0.092	1.172
7cjm _sitemap_1_site_5	0.579	27	0.509	60.711	0.649	0.616	1.024
7m1y							
7m1y_sitemap_1_site_1	0.650	35	0.599	104.272	0.715	0.213	0.986
7m1y_sitemap_1_site_2	0.745	36	0.517	151.949	0.550	0.015	1.535
7m1y_sitemap_1_site_3	0.696	38	0.658	128.625	0.750	0.284	0.913
7m1y_sitemap_1_site_4	0.667	22	0.595	56.595	0.577	0.850	0.884
7ofs							
7ofs_sitemap_1_site_1	1.154	87	1.240	131.369	0.356	3.081	0.378
7ofs_sitemap_1_site_2	0.929	75	0.925	206.143	0.559	0.501	0.992
7ofs_sitemap_1_site_3	0.885	71	0.730	142.345	0.486	0.107	1.478
7ofs_sitemap_1_site_4	0.701	42	0.675	129.654	0.734	0.341	0.892
7ofs_sitemap_1_site_5	0.656	38	0.624	114.219	0.752	0.209	0.900
7d47							
7d47_sitemap_1_site_1	0.779	60	0.759	166.698	0.726	0.205	1.027
7d47_sitemap_1_site_2	0.808	57	0.667	146.804	0.581	0.055	1.381
7d47_sitemap_1_site_3	0.780	42	0.785	69.286	0.571	1.066	0.632
7d47_sitemap_1_site_4	0.658	35	0.589	105.987	0.650	0.137	1.065
7d47_sitemap_1_site_5	0.615	30	0.610	39.102	0.703	0.416	0.625
7d6h							
7d6h_sitemap_1_site_1	0.819	61	0.732	159.495	0.545	0.081	1.236
7d6h_sitemap_1_site_2	0.895	59	0.945	92.953	0.487	1.585	0.491
7d6h_sitemap_1_site_3	0.678	47	0.589	73.059	0.618	0.008	1.204
7d6h_sitemap_1_site_4	0.672	37	0.605	98.784	0.619	0.138	1.073
7d6h_sitemap_1_site_5	0.786	39	0.812	112.847	0.721	0.978	0.448

Table S2. Site detection information of Cavity

Entry Name	RankScore	DrugScore	Hydrophobic	Acceptor	Donor	volume (A^3)
6wuu						
vacant1	10.447360	-162	121	1236	1688	779.000
vacant2	7.7925640	35	87	834	1638	774.375
vacant3	7.7517430	-281	86	433	835	458.000
vacant4	7.0805280	-1321	15	534	797	430.375
vacant5	6.0967800	-300	167	857	1023	633.125
7cjm						
vacant1	8.5211300	-850	125	1943	2211	446.625
vacant2	8.2056350	-639	433	2464	3385	754.625
vacant3	8.0488300	-1045	435	1056	2082	547.375
vacant4	7.8262500	-620	668	1535	2320	468.625
vacant5	7.2431290	409	1354	2114	4357	773.500
7ofs						
vacant1	8.0402660	-834	763	1261	2138	653.000
vacant2	7.7967570	-565	673	1683	2580	506.000
vacant3	7.0425050	-735	257	2145	2683	517.125
vacant4	6.8945670	-1019	234	1288	2149	482.250
vacant5	6.8386200	609	1806	2127	3671	816.125
7d47						
vacant1	8.8970820	-704	239	2666	3418	710.375
vacant2	7.5544520	-772	365	1599	2734	528.250
vacant3	7.0460890	-515	722	1526	2400	660.125
vacant4	6.7442840	-840	743	665	1460	332.000
vacant5	6.5531780	-226	1287	1346	2476	616.125
7d6h						
vacant1	10.134752	-179	1444	1601	3263	856.000
vacant2	7.9764980	-551	791	1847	3447	826.375
vacant3	7.8480600	-645	348	2645	3442	744.375
vacant4	7.7824750	-758	433	1651	2887	557.625
vacant5	7.3167430	42	1526	1993	3119	776.625
7m1y						
vacant1	9.3610900	-622	262	2118	2253	462.750
vacant2	9.3040700	-133	1583	1668	3303	873.125
vacant3	8.2173920	-334	860	1308	2743	619.250
vacant4	8.2030000	-489	786	1724	2309	502.250
vacant5	6.5381150	-686	659	1063	1505	329.625

Table S3. Site detection information of DeepSite

Entry Name	scores	x	y	z
6wuu				
1	0.97285	63.56	71.26	85.41
2	0.96931	85.56	47.26	63.41
3	0.98226	77.56	91.26	81.41
4	0.83317	75.56	51.26	77.41
5	0.42006	85.56	65.26	83.41
7cjm				
1	0.98817	64.99	65.67	74.43
2	0.91699	78.99	91.67	82.43
3	0.88224	76.99	55.67	54.43
7d6h				
1	0.94939	63.98	67.84	73.28
2	0.94141	79.98	91.84	83.28
3	0.86360	73.98	53.84	51.28
4	0.88239	87.98	77.84	81.28
7d47				
1	0.89974	63.14	67.38	74.95
2	0.84649	79.14	51.38	78.95
3	0.83823	73.14	57.38	60.95
4	0.88938	81.14	93.38	82.95
5	0.63018	79.14	95.38	64.95
7m1y				
1	0.94182	81.28	93.59	81.69
2	0.88745	75.28	51.59	77.69
3	0.93150	63.28	67.59	73.69
4	0.76464	75.28	55.59	55.69
5	0.71294	73.28	81.59	67.69
7ofs				
1	0.99315	76.85	95.48	67.19
2	0.98623	64.85	67.48	73.19
3	0.77673	72.85	51.48	77.19
4	0.83836	72.85	55.48	53.19
5	0.81327	88.85	77.48	77.19
6	0.56932	66.85	73.48	89.19

Figure S1. The contribution of each residue to the pocket boundary in trajectories.

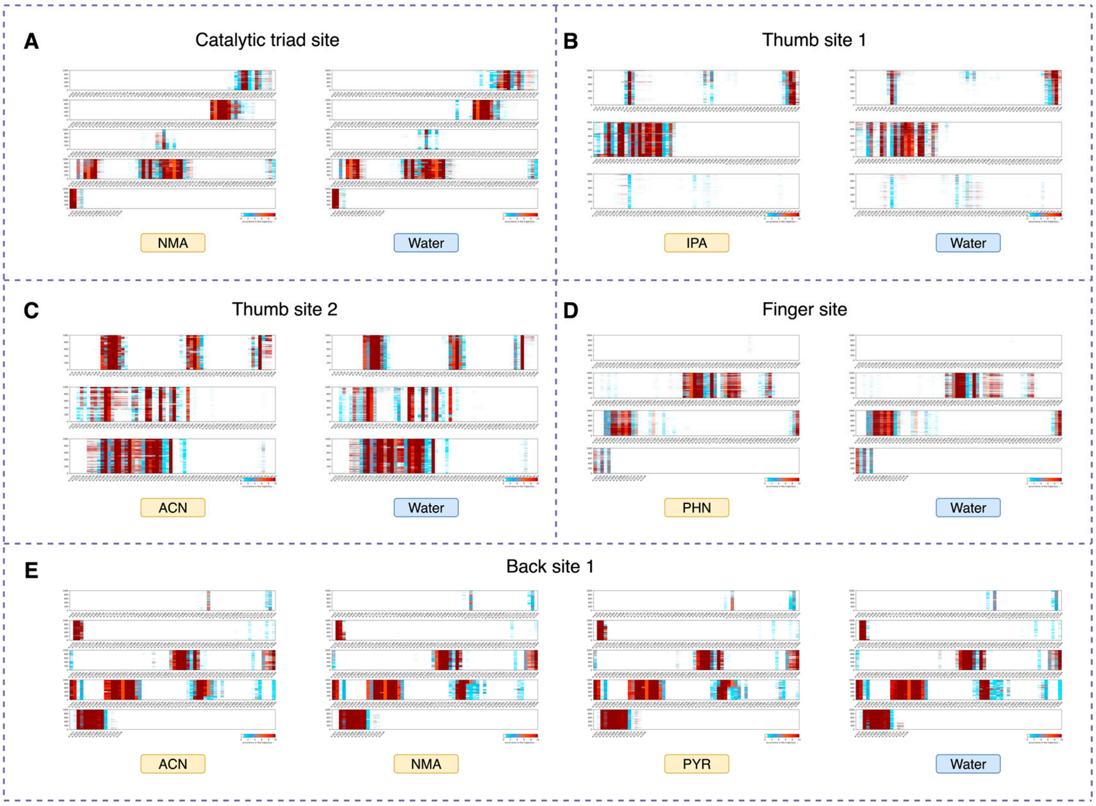

Figure S1. The contribution of each residue to the pocket boundary in trajectories. (A) For catalytic triad sites, NMA MixMD system and pure water system. (B) For thumb site 1, IPA MixMD system and pure water system. (C) For Thumb site 2, ACN MixMD system and pure water system. (D) For finger site, PHN MixMD system and pure water system. (E) For back site 1, from left to right, it corresponds to ACN, NMA, PYR MixMD system and pure water system respectively.

Figure S2. The distribution of clusters.

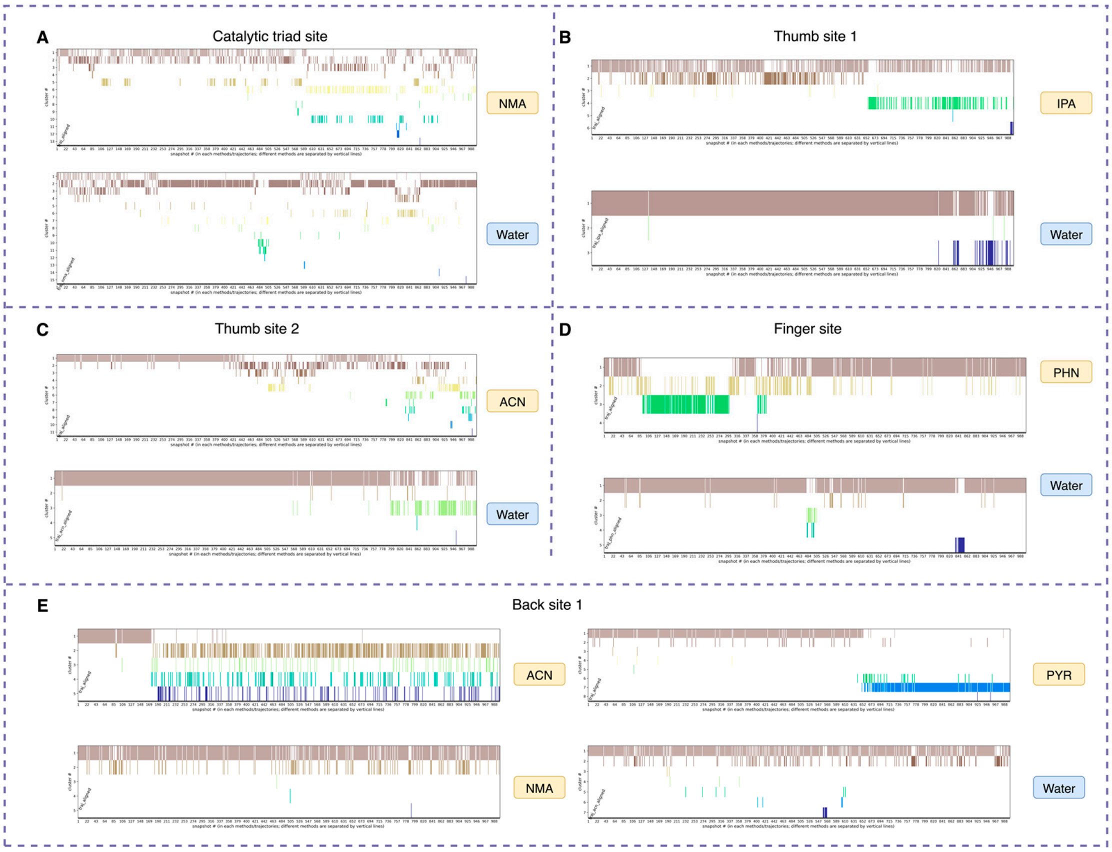

Figure S2. The distribution of clusters. (A) For catalytic triad sites, NMA MixMD system and pure water system. (B) For thumb site 1, IPA MixMD system and pure water system. (C) For Thumb site 2, ACN MixMD system and pure water system. (D) For finger site, PHN MixMD system and pure water system. (E) For back site 1, ACN, NMA, PYR MixMD system and pure water system respectively.

Figure S3. Physicochemical properties of the pocket along the MD trajectory of the clustering result.

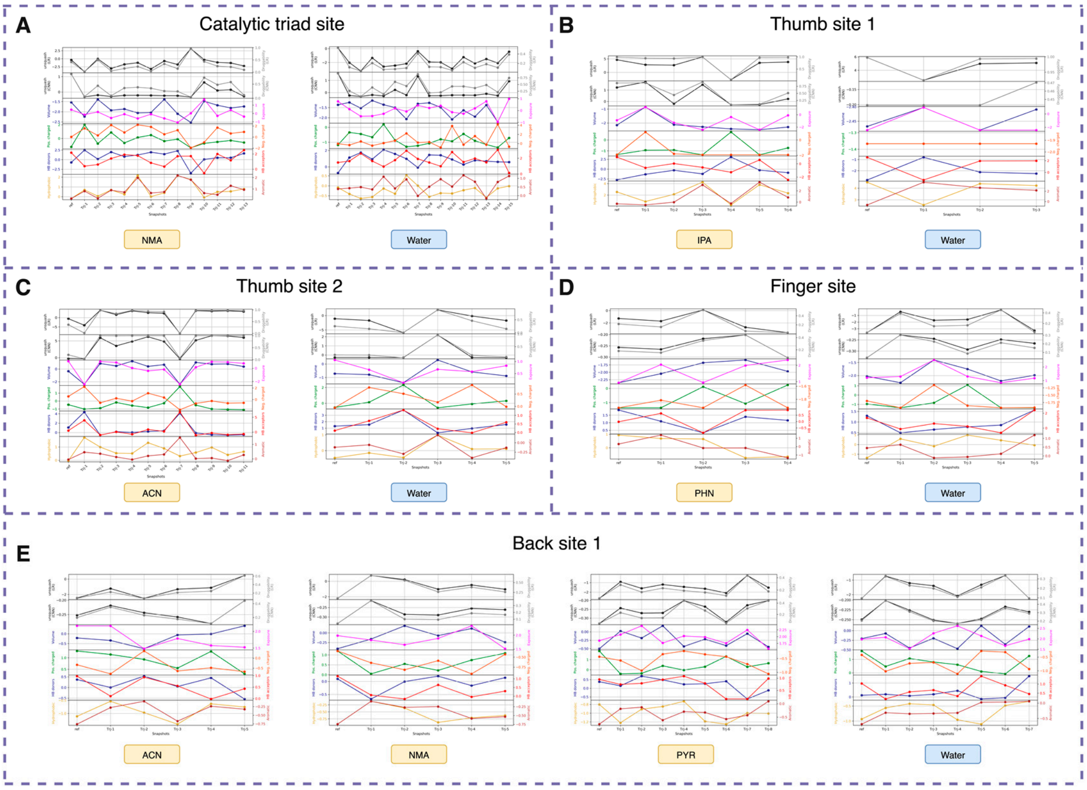

Figure S3. Physicochemical properties of the pocket along the MD trajectory of the clustering result. color defines the number of heavy atoms in each residue that contribute to the pocket boundary in a par- 418 ticular snapshot. The darker the color, the closer the residue relates to the pocket. (A) For catalytic triad sites, NMA MixMD system and pure water system. (B) For thumb site 1, IPA MixMD system and pure water system. (C) For Thumb site 2, ACN MixMD system and pure water system. (D) For finger site, PHN MixMD system and pure water system. (E) For back site 1, from left to right, it corresponds to ACN, NMA, PYR MixMD system and pure water system respectively.

Figure S4. The heatmap for the TRAPP-LR/CNN models for selected specific cluster.

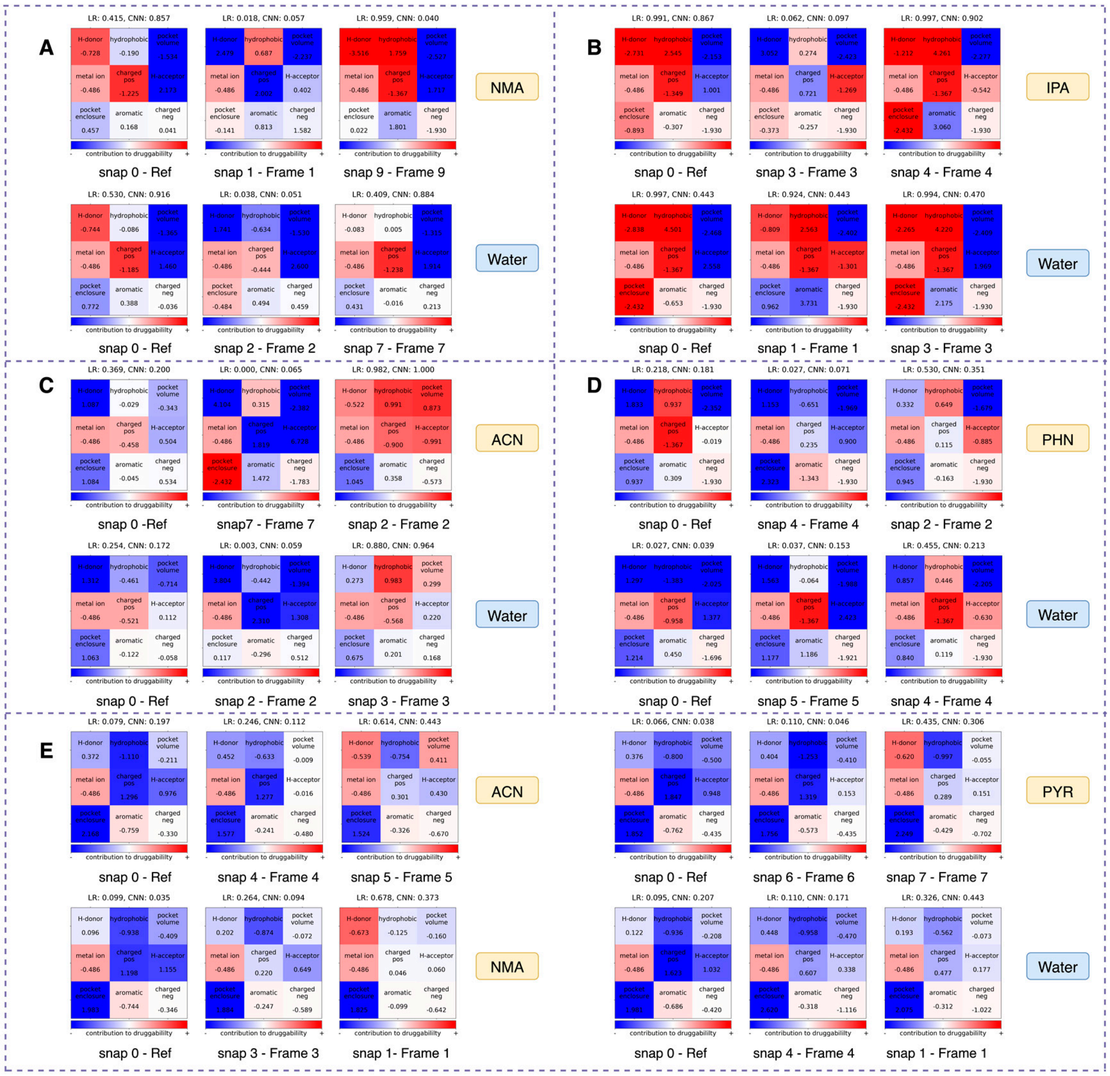

Figure S4. The heatmap for the TRAPP-LR/CNN models for selected specific cluster. Each row from left to right corresponds to the reference conformation, the cluster conformation with lowest druggability score and the cluster conformation with relative highest druggability score. (A) For catalytic triad sites, NMA MixMD system and pure water system. (B) For thumb site 1, IPA MixMD system and pure water system. (C) For Thumb site 2, ACN MixMD system and pure water system. (D) For finger site, PHN MixMD system and pure water system. (E) For back site 1, ACN, NMA, PYR MixMD system and pure water system respectively.

Figure S5. The pocket conformation for specific cluster.

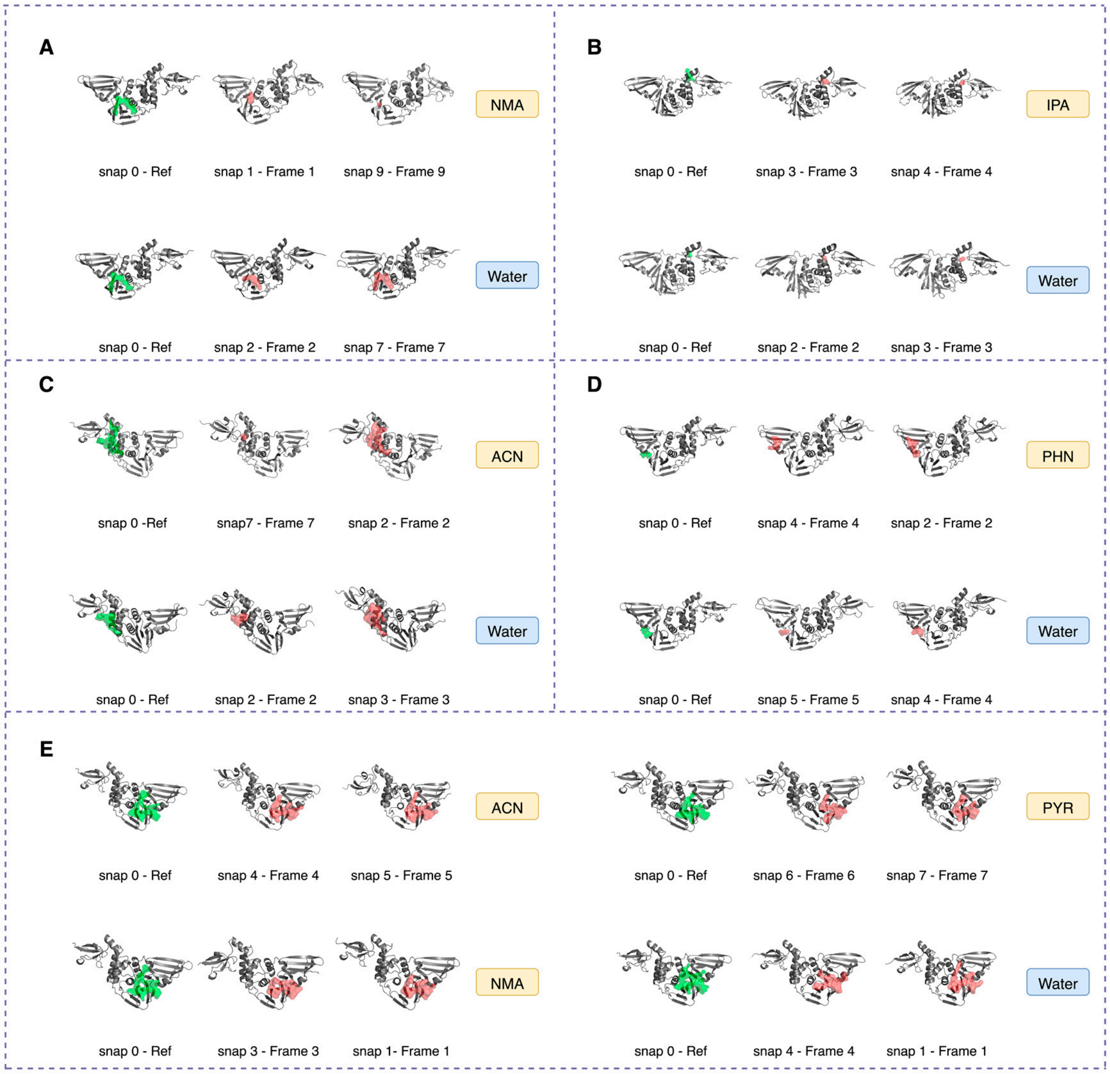

Figure S5. The pocket conformation for specific cluster. The pocket distribution is shown in green and red. Each row from left to right corresponds to the reference conformation, the cluster conformation with lowest druggability score and the cluster conformation with relative highest druggability score. (A) For catalytic triad sites, NMA MixMD system and pure water system. (B) For thumb site 1, IPA MixMD system and pure water system. (C) For Thumb site 2, ACN MixMD system and pure water system. (D) For finger site, PHN MixMD system and pure water system. (E) For back site 1, ACN, NMA, PYR MixMD system and pure water system respectively.
